# Feeders of Free-Roaming Cats: Personal Characteristics, Feeding Practices, and Data on Cat Health and Welfare in an Urban Setting of Israel

**DOI:** 10.3389/fvets.2016.00021

**Published:** 2016-03-07

**Authors:** Idit Gunther, Tal Raz, Yehonatan Even Zor, Yuval Bachowski, Eyal Klement

**Affiliations:** ^1^Koret School of Veterinary Medicine, Hebrew University of Jerusalem, Jerusalem, Israel; ^2^Veterinary Municipal Department, Rishon-Lezion, Israel

**Keywords:** cat feeders, cat caretakers, free-roaming cats, neutering, feeding habits, TNR, sterilization

## Abstract

Cat feeders serve as an important source of available food for free-roaming cats (FRCs) and can play a central role in providing data on FRC distribution, welfare, and health. Data on cat feeder personalities as well as a better understanding of their feeding practices offer relevance for decision making concerning FRC population control strategies. The current study surveyed 222 FRC feeders who responded to a municipal trap-neuter-return (TNR) campaign in an Israeli central urban setting. The aim of the study was to describe their personal characteristics, feeding practices, and the FRC populations they feed. Feeders were divided into four groups according to the number of cats they claimed to feed per day (group 1: fed up to 5 cats, group 2: fed 6–10 cats, group 3: fed 11–20 cats, and group 4: fed ≥21 cats). Most feeders were women (81%), with a median age of 58 years (range 18–81). The feeders reported an overall feeding of 3337 cats in 342 different feeding locations. Feeders of group 4 comprised 15.31% (*n* = 34) of all feeders but fed 56% (*n* = 1869) of the FRC in 37.42% (*n* = 128) of the feeding locations. “Heavy” feeders (groups 3 and 4) reported that they traveled significantly longer distances in order to feed the cats. Commercial dry food consisted of 90% of the food they provided, with 66% of them feeding once a day, with less food per cat per day than the other feeder groups. Interestingly, “heavy” feeders were usually singles, had on average fewer offspring, a clear preference for owning cats as pets, and lived in lower income neighborhoods. According to the feeders’ reports on the FRC populations they fed, 69.7% (2325/3337) cats were neutered and 11.8% (395/3337) were kittens. In addition, they reported that 1.6% (54/3337) of the cats were limping, 2% (67/3337) suffered from a systemic disease, 4% (135/3337) had skin lesions, and 3.9% (130/3337) were suffering from a chronic disability. Abundance of kittens and morbidity rate were significantly and negatively associated with neutering rate. These findings are in accordance with the suggestion that neutering may potentially improve cat welfare by reducing morbidity. Collaboration by the authorities with these heavy feeders, who represent a small number of FRC feeders and feed substantial FRC numbers, may be significant for the control and monitoring of FRC populations and their resources.

## Introduction

Free-roaming cats (FRCs) living in close proximity to humans might constitute a potential public health risk and cause nuisances. Their potential to transmit several zoonotic diseases (e.g., toxoplasmosis, rabies, cat scratch disease, Q fever, toxocariasis, and flea-borne typhus), has been well described in the literature (1–4). Their potential nuisance aspects (e.g., hygiene issues, aggressiveness toward people, and invasion of human facilities) and welfare impairment have only recently been examined and measured (5). Furthermore, cat predation on wildlife has an extreme influence on biodiversity not only in natural areas but also in urban and rural areas (6, 7).

In Israel, active feeding of FRC provides an important food source for these cats, especially in urban settings (8). Feeding FRC has been previously described in other countries, such as Italy (9) and the USA (10). However, a high variation in number of feeders in the local community is expected due to cultural and climate differences. Several studies have evaluated the role of cat feeders in local communities, mainly in the USA. Haspel and Calhoon (11) randomly surveyed households in Brooklyn and estimated a rate of 28 daily cat feeders per 1000 citizens. Their estimated rate of occasional feeders (feeding between once a week to five times a year) was significantly higher, and reaching 192 feeders per 1000 citizens. In another random household survey performed in Florida, researchers found a rate of 4.3 daily feeders and a rate of 76.7 occasional feeders per 1000 citizens. This proportion of cat feeders is compatible with another study performed in the USA (10). The role of cat feeders in maintaining FRC populations is further emphasized when taking into consideration the findings of a few additional studies and unofficial reports from the USA, which estimated a mean number of 3.3 feeding FRC per feeder (10).

Despite the high abundance of cat feeders worldwide, there are only limited data available on their personal characteristics and motivation. In one study, most daily feeders were women aged 50–79 years (11), while in another study, a median age of 45 years was found (12). It was also suggested that in comparison to non-feeders, cat feeders are more likely to own pet cats (10–12). Haspel and Calhoon (11) and Natoli et al. (9) found that the cat feeders delivered higher quantities of food than their demanded by physiological needs of the fed cats in the studied areas. Feeders also held the perception that the cats were hungry. Consequently, the researchers deduced that cat feeding fulfills the carers’ need for nurturing and contributes to the quality of city life by providing companionship to these carers.

The use of culling for controlling FRC populations in Israel was banned by the Israeli Supreme Court in 2004. Since then, the method of trap-neuter-return (TNR) is frequently used for controlling FRC populations and managing their associated nuisances. In addition, it was previously suggested that neutering might improve cat health and thereby contribute to their welfare (13–15). Local municipalities and the Israeli Ministry of Agriculture and Rural Development invest millions of dollars in TNR actions each year. Most of these actions are performed with the collaboration of FRC feeders, who continually request neutering activities from the municipalities (personal communication with the authorities). This enabled us to contact and track the FRC feeders in order to characterize them, as well as the health status of the FRC they feed. Such description of FRC feeder characteristics and their feeding practices may help in determining strategies for the control of FRC populations and their welfare. Such control strategies might incorporate the assistance of cat feeders for controlling the distribution of cat food resources, for monitoring cat populations during management efforts, and even for aiding in the capture of FRC.

Hence, the study objectives were (1) to describe the feeding practices of FRC feeders, (2) to describe the personal characteristics of FRC feeders, (3) to determine possible associations between feeders’ personal characteristics and their feeding practices, and (4) to determine the association between reported neutering rates and health and welfare conditions of FRC.

## Materials and Methods

### Study Population and Data Collection

The study population comprised cat feeders who were registered with the municipal veterinary services of the city of Rishon-Lezion, Israel. The total population of Rishon-Lezion was 237,600 citizens at the end of 2013 (Central Bureau of Statistics, Israel). Data consisted of all requests to the municipality for FRC trap-neuter-return (TNR) by cat feeders, registered with the municipal veterinary services between February 2010 and October 2013. These requests followed municipal advertisements in the local press, encouraging citizens to report on preferred locations for municipal TNR actions to take place. The respondents were requested to provide information on location and neighborhood for the TNR requested actions, number of cats to be neutered, date of application, name of applicant, and applicant’s phone number. In addition, for each request, the applicants were asked if they consider themselves as cat feeders. Of the total request list, double applications and non-feeders were excluded from further consideration. This dataset was the basis for a cross-sectional telephone interview study, which was performed in the Hebrew language by one of the authors (Yuval Bachowski), between July 16 and October 20, 2013. Calls were made at different times of the day and on different days. This enabled maximal opportunity to contact the applicants, aiming to contact as much feeders listed in the dataset. At least three attempts were made to contact each applicant. Duration of interviews varied between 5 and 30 min, depending on the number of fed cats and feeding locations.

### Questionnaire Contents

The questionnaire was developed based on the study aims and was validated by a pilot telephone survey to cat feeders that fed cats in another city (*n* = 10). A total of 16 questions were divided into three sections related to the following issues: (1) feeding practices, (2) description of the feeding cats, and (3) personal details of the feeder. Questionnaire is provided in the supplementary information. Cat feeders were asked first for the locations and frequency of feeding, type and amount of food they provide, number of cats they feed in each feeding group, and whether they routinely also provide water to the FRC. They were then asked to provide their most accurate estimation of the number of kittens (up to 6 months old), neutered cats (marked in Israel by ear tipping), cats with systemic diseases (diminished cat function, such as apathy, anorexia, and acute exercise intolerance), skin lesions, and permanent disability (such as eye absence and limb deformation or absence), among the FRC they had fed during the 2 weeks prior to filling in the questionnaire. Finally, cat feeders were asked for their year of birth, home address, marital status, number of offspring, number of offspring living with them, and for the species and number of their pets.

The study was approved by the Hebrew University Institutional Ethical Committee. Consent procedure and subject confidentiality were strictly implemented.

### Socioeconomic Regional Data

Data on feeders’ neighborhoods were obtained from the 2008 population census of the Israeli Bureau of Statistics. For this census, the State of Israel was divided into 1616 geographic areas, called “statistical areas.” These areas were determined according to spatial features and roughly according to homogeneity of socioeconomic characteristics. The city of Rishon-Lezion was divided into a total of 63 statistical areas, of which 58 were residential areas with an average population of 3000 citizens each. The statistical areas were graded according to socioeconomic status, calculated using multiple criteria (grades ranged from 1 to 1616 of the total statistical areas of Israel; www.cbs.gov.il). Further data for each statistical area included education level of citizens aged 25–54 (presented as the average number of years of education), percentage of people aged 25–54 who possessed an academic degree, and average income per citizen [in New Israel Shekels (NIS)].

Age and gender distribution of the general adult population of the district of Rishon-Lezion were obtained from the 2013 data of the Israeli Bureau of Statistics and truncated to exclude the population below 20 years of age (in order to be comparable with the present study, which incorporated only people older than 19 years).

### Statistical Analyses

Personal characteristics of feeders, their feeding practices, and the details of the cats they fed were summarized on an Excel datasheet. Spatial distribution of feeders’ home addresses and feeding locations were summarized using Arcmap 10.2.1. Analyses were performed using either SPSS 21.0^®^ for Windows^®^ or WinPepi version 4.0 (2007). In all analyses, a significance level of *p* < 0.05 was applied.

#### Feeding Practices and Personal Characteristics

In order to examine trend association of feeding practices and personal characteristics in relation with the number of fed FRC per feeder, cat feeders were arbitrarily divided into four groups according to the total number of cats they had claimed to feed per day: (1) fed up to 5 cats, (2) fed 6–10 cats, (3) fed 11–20 cats, and (4) fed 21 cats and more. These ranks are referred to below as feeder groups 1–4, respectively.

For categorical data, χ^2^ for trend was performed to examine trend among feeder groups 1–4. For continuous data, we first visualized Q–Q plot for normal distribution and then performed Leven’s test for homogeneity of variance. To examine trend among groups 1–4 of variables with normal distribution and similar variances between groups, a Pearson correlation coefficient was calculated. For all other cases, a non-parametric Spearman correlation coefficient was calculated for trend. For analyzing distance between home address to feeding locations, data of five feeders whom home address were out of the city of Rishon-Lezion were omitted.

Age distribution of the feeders was compared to age distribution of the general adult population, using independent samples *T*-test. Gender differences between the feeder population and the general adult population of the district were compared, using chi-square test.

#### Neutering and Cat Health

Association between neutering rate of FRC per feeder and the presence of kittens and health status of the cats per feeder was determined by fitting a binary logistic regression model to each of the dependent variables separately (kittens, limping, systemic disease, skin lesions, and chronic disability), controlling for feeder’s group rank. This analysis was performed only for feeding groups of whose size exceeded five cats.

## Results

A total of 1092 TNR requests were registered at the Veterinary Services of the city of Rishon-Lezion between February 2010 and October 2013. A total of 364 FRC feeders were identified in these requests. Of these, 142 feeders did not participate in the survey: 5 due to language barrier, 29 refused, 46 were unavailable despite three phone calls, 52 were no longer FRC feeders, 9 had left the city, and 1 was deceased. Not all respondents answered all the questions, due to differences in their willingness to share personal data.

### Feeding Practices

**Figure 1:**
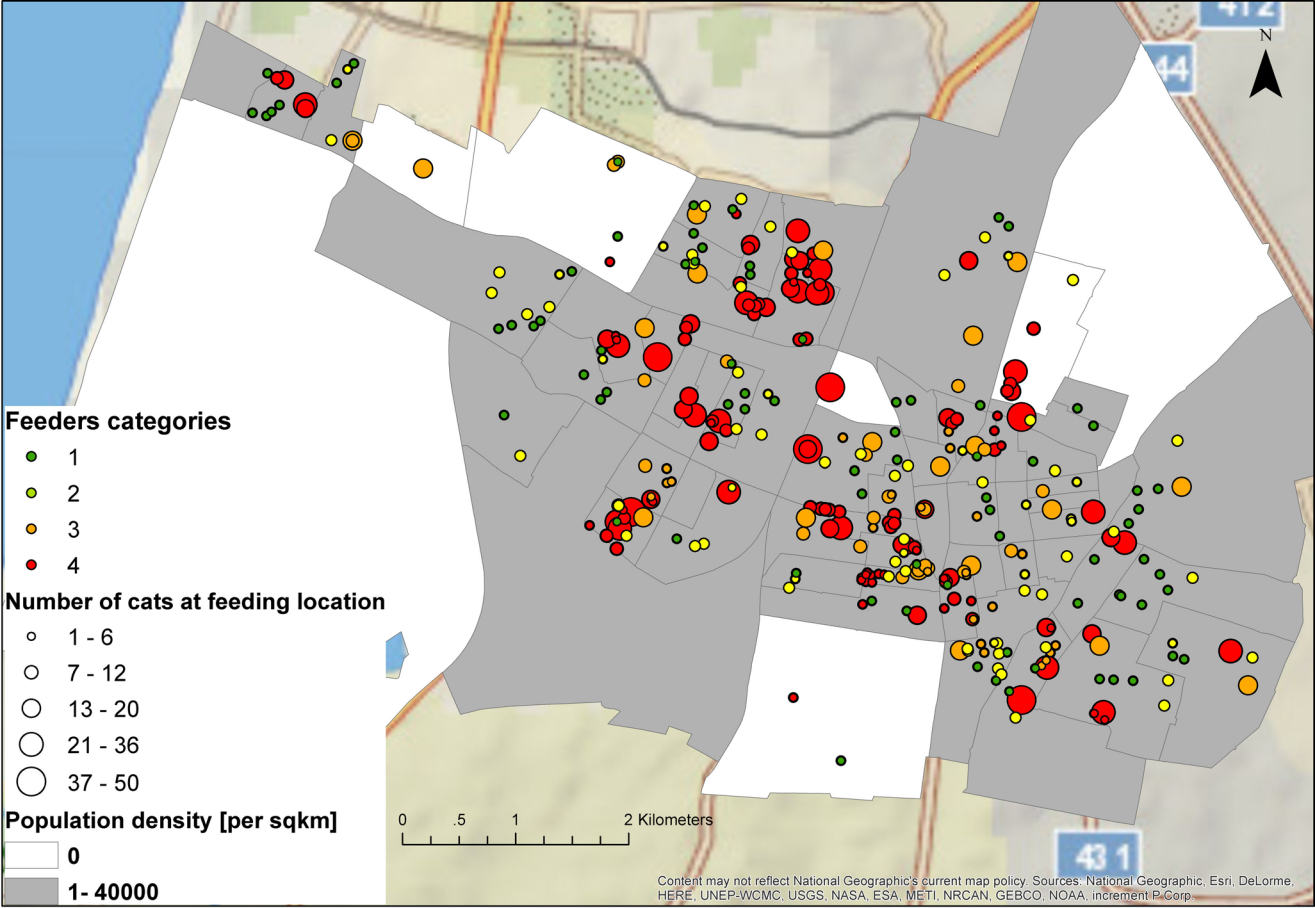
**Free-roaming cat (FRC) feeding locations in 58 statistical areas of the city of Rishon-Lezion, Israel**. The color of the statistical areas represents residential areas (grey) and non-residential areas (white). Each feeding location represents a feeder category according to the total number of FRC that each feeder feeds (point color; green – feed 1–5 FRC, yellow – feed 6–10 FRC, orange – feed 11–20 FRC, and red – feed ≥21 FRC) and to the number of fed cats in each location (point size).

The study population comprised 222 feeders who reported feeding a total of 3337 cats in 342 different feeding locations in the city of Rishon-Lezion. Feeders in group 4 comprised 15.3% (*n* = 34) of all feeders but fed 56% (*n* = 1869) of the FRC in 37.4% (*n* = 128) of the feeding locations (Table 1; Figure 1). Eleven feeders in group 4 fed ≥50 FRC each (up to 320 cats per feeder). These 11 feeders (5% of all feeders) fed a total of 1103 FRC constituting 33% of all fed FRC in this survey; of these, two feeders fed 541 (16.2%) of the total reported fed FRC.

**Table 1:** **Feeding practices of 222 free-roaming cat (FRC) feeders surveyed in the city of Rishon-Lezion, Israel during 2013 (group 1: fed up to 5 cats, group 2: fed 6–10 cats, group 3: fed 11–20 cats, and group 4: fed ≥21 cats)**.

	Group 1	Group 2	Group 3	Group 4	Correlation coefficient	*p*-value for trend among groups 1–4
**General information**						
Total of feeders	78	67	43	34	NA	NA
Total of FRC fed	258	563	647	1869	NA	NA
Average no. of cats per feeder (mean ± SD)	3.3 ± 1.4	8.4 ± 1.6	15 ± 3.3	54.9 ± 58.1	NA	NA
**Feeding locations**						
Total number of feeding locations	79	71	64	128	NA	NA
Average feeding locations per feeder (mean ± SD)	1 ± 0.1	1.1 ± 0.2	1.5 ± 0.9	3.8 ± 3.2	NA	NA
Distance (m) from feeder’s home to feeding location (mean ± SD)	109 ± 669 (*n* = 77)	24 ± 100 (*n* = 69)	73 ± 161 (*n* = 60)	413 ± 849 (*n* = 128)	0.62	<0.001^b^
**Food types and quantities**						
Dry food per cat per day (g; mean ± SD)	62 ± 60 (*n* = 70)	55 ± 40 (*n* = 64)	50 ± 24 (*n* = 42)	38 ± 27 (*n* = 30)	−0.13	0.074^b^
Moist food per cat per day (g^c^; mean ± SD)	2.2 ± 8.3 (*n* = 77)	1.8 ± 6.0 (*n* = 67)	3.0 ± 9.0 (*n* = 43)	1.2 ± 2.2 (*n* = 31)	0.18	0.008^b^
Leftovers per cat per day (g; mean ± SD)	23 ± 54.9 (*n* = 64)	8.9 ± 25.8 (*n* = 57)	3 ± 8.7 (*n* = 40)	1.7 ± 8.4 (*n* = 32)	−0.25	0.001^b^
Proportion of locations in which water is supplied (%)	77.2 (61/79)	81.7 (58/71)	82.8 (53/64)	71.2 (89/125)	NA	0.157^a^
**Feeding frequency**						
Daily feeders (rank 0 or 1^d^; mean ± SD)	0.85 (*n* = 78)	0.88 (*n* = 67)	0.98 (*n* = 43)	0.97 (*n* = 34)	0.17	0.009^b^

*NA, not applicable (could not be performed or directly related to group division)*.

*^a^χ^2^ test for trend*.

*^b^Spearman’s correlation test*.

*^c^Data were originally collected in units of “cans per cat per day” and transformed to grams taking into account an average weight of 156 g per can*.

*^d^Rank 0 for a non-daily feeder and 1 for a daily feeder*.

**Figure 2:**
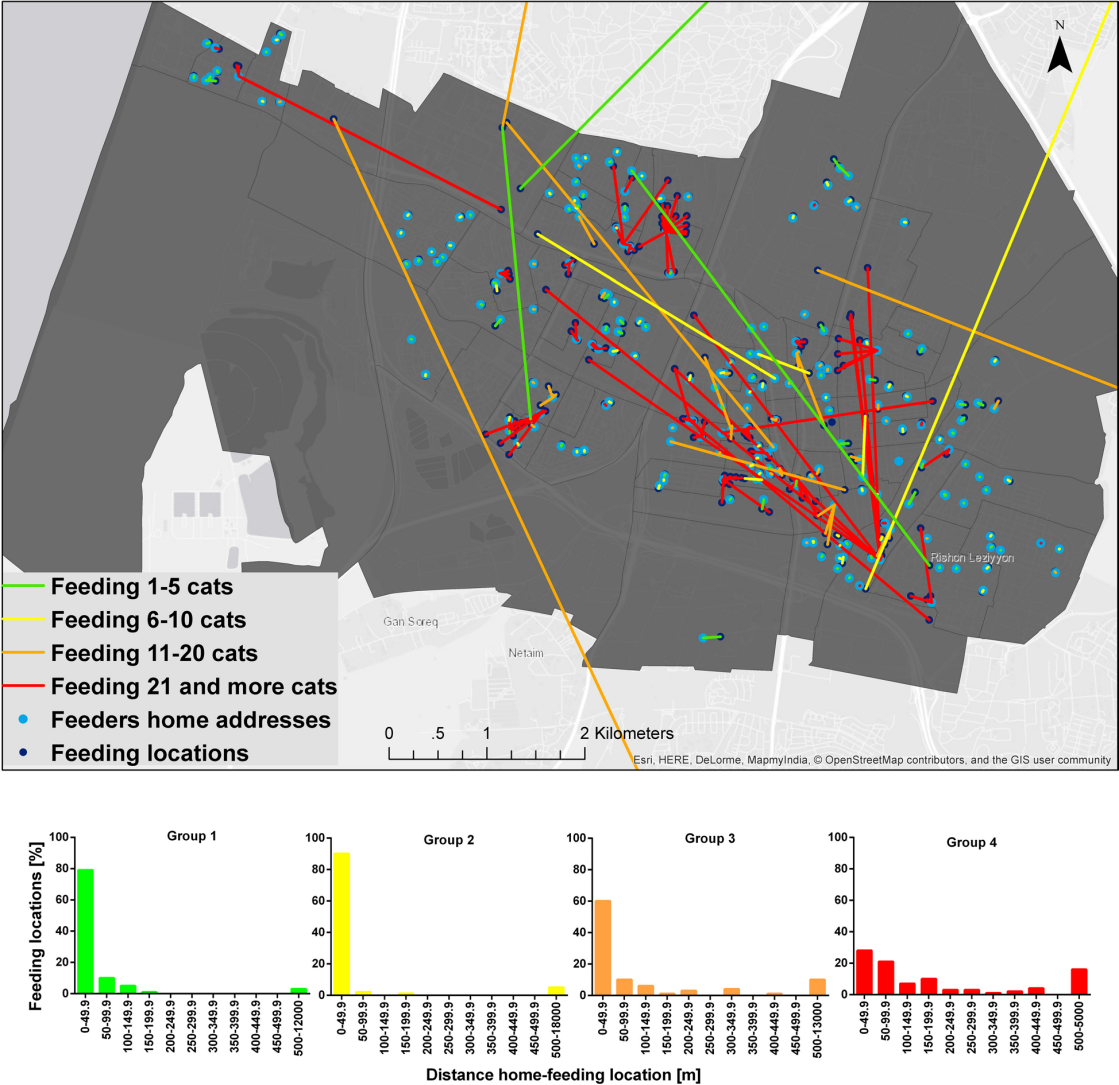
**Distances from feeders’ home to feeding locations of free-roaming cats (FRC) in the city of Rishon-Lezion**. The color of each line represents a feeder category according to the total number of FRC that each feeder feeds (green – feed 1–5 FRC, yellow – feed 6–10 FRC, orange – feed 11–20 FRC, and red – feed ≥21 FRC). A frequency distribution graph is provided of these distances among each feeder group.

Feeders of group 4 traveled on average the longest distance from their home to feeding locations (Table 1; Figure 2). Median distance for groups 1–3 was 0 while for group 4, it was 79 m.

There was no significant difference between groups in the proportion of feeders who provided water to the FRC. Feeders fed mainly dry commercial food, followed by leftovers, and with a minority feeding moist canned commercial food. The amount of leftovers was negatively associated with feeder group category. The total amount of food delivered per cat decreased gradually with feeder group: feeders in group 1 delivered the largest amount of food and feeders in group 4 the smallest. Two hundred feeders (90%) reported feeding FRC on a daily basis. The proportion of these daily feeders was significantly associated with feeder group category (Table 1).

### Personal Characteristics

Most feeders (180/222, 81.1%) were women, with almost no difference in this sex ratio among the four feeder groups. This sex ratio is much higher than the percentage of women in the general adult population in Rishon-Lezion (50.84%, chi-square, *p* < 0.001). Feeder age distribution (median: 58 years, mean ± SD: 55 ± 13.8 years, with almost no difference among the four feeder groups) was higher than the age of the general adult population (median: 44.7 years, mean ± SD: 46.3 ± 0.2 years, *p* < 0.001). Overall, most feeders were married (153/215, 71.2%), while the rest (62/215, 28.8%) were single, divorced, or widows. Most feeders had offspring (175/213, 82.1%), but 37.7% of them (66/175) were no longer living with their offspring during the survey period. Most feeders owned pets (158/220, 71.8%), 65.2% (103/220) owned pet cats (in addition to the FRCs they fed), 54.4% (86/220) owned dogs, and 7.59% (12/220) owned other pets. Due to the small number of feeders who owned pets other than cats and dogs, their data were not included in further statistical analysis of pet ownership among feeders.

In comparison to “light” feeders (groups 1–2), significantly more of the “heavy” feeders (groups 3–4) were not married, did not have offspring, did not live with their offspring, and owned pets, mainly cats. No significant differences were found in the percentage of dog ownership or the average number of pet dogs owned (Table 2).

**Table 2:** **Personal characteristics of 222 free-roaming cat (FRC) feeders surveyed in the city of Rishon-Lezion, Israel during 2013 (group 1: fed up to 5 cats, group 2: fed 6–10 cats, group 3: fed 11–20 cats, and group 4: fed ≥21 cats)**.

	Group 1	Group 2	Group 3	Group 4	Correlation coefficient	*p*-value for trend among groups 1–4
**General information**						
Total of feeders	78	67	43	34	NA	NA
Proportion of women (%)	76.9 (60/78)	82.1 (55/67)	81.4 (35/43)	88.2 (30/34)	NA	0.186^b^
Age (*n* = 207) (median ± SD; range)	59.5 ± 13.6 (27–81)	58.5 ± 14.1 (20–79)	56 ± 12.7 (21–79)	58.5 ± 14.8 (18–79)	−0.05	0.508^c^
**Familial status**						
Proportion feeders who are not married (%)^a^ (*n* = 215)	9.2 (7/76)	27.7 (18/65)	40.5 (17/42)	62.5 (20/32)	NA	<0.001^b^
Proportion of feeders without offspring (%) (*n* = 213)	6.8 (5/73)	18.5 (12/65)	21.4 (9/42)	36.4 (12/33)	NA	<0.001^b^
Number of offspring living with feeder (mean ± SD)	1.2 ± 1.28	1.25 ± 1.2	0.84 ± 0.76	0.7 ± 0.86	−0.15	0.022^d^
**Pets**						
Proportion of pet-owning feeders (%) (*n* = 220)	58.4 (45/77)	71.2 (47/66)	81.4 (35/43)	91.2 (31/34)	NA	<0.001^b^
Proportion of pet-owning feeders who own pet cats (%) (*n* = 158)	37.8 (17/45)	68.1 (32/47)	71.4 (25/35)	93.5 (29/31)	NA	<0.001^b^
Number of pet cats (mean ± SD)	0.3 ± 0.6	0.9 ± 1	1.5 ± 1.9	2.8 ± 2.7	0.47	<0.001^d^
Proportion of pet-owning feeders who own pet dogs (%) (*n* = 158)	62.2 (28/45)	44.7 (21/47)	57.1 (20/35)	54.8 (17/31)	NA	0.1^b^
Number of dogs (mean ± SD)	0.4 ± 0.7	0.4 ± 0.7	0.6 ± 0.8	0.6 ± 0.7	0.10	0.134^d^

*NA, not applicable (could not be performed or directly related to group division)*.

*^a^Single, divorced, or widow*.

*^b^χ^2^ test for trend*.

*^c^Pearson correlation coefficient test*.

*^d^Spearman’s correlation test*.

No trend association was found between feeder group and socioeconomic parameters in the residential neighborhoods (Table 3). However, a significant difference was found between “light” feeders (groups 1–2) and “heavy” feeders (groups 3–4) in regard to the average income in their residential neighborhoods (*p* < 0.05); “heavy” feeders lived in neighborhoods with lower income. The number of feeders per 1000 citizens was not associated with residential neighborhood average income (*r* = 0.17, *p* = 0.22).

**Table 3:** **Comparison of socioeconomic neighborhood (statistical areas) characteristics of 222 free-roaming cat (FRC) feeders surveyed in the city of Rishon-Lezion, Israel during 2013**.

	Group 1	Group 2	Group 3	Group 4	Correlation coefficient	*p*-value for trend among groups 1–4
Socioeconomic status grade (mean ± SD)	1091 ± 266	1099 ± 262	1064 ± 241	1068 ± 258	−0.04	0.605^a^
Years of education (age 25–54) (mean ± SD)	13.8 ± 1	13.7 ± 1.1	13.3 ± 2.5	13.6 ± 1	−0.06	0.403^a^
Percentage of academic degree (age 25–54) (mean ± SD)	32.1 ± 10.3	31.8 ± 9.5	30.2 ± 10.4	29.6 ± 8.4	−0.09	0.207^a^
Average income per person ($; mean ± SD)	1781 ± 436	1776 ± 465	1568 ± 419	1663 ± 367	−0.12	0.088^b^

*Feeders were divided to four groups: group 1: fed up to 5 cats, group 2: fed 6–10 cats, group 3: fed 11–20 cats, and group 4: fed ≥21 cats*.

*^a^Pearson correlation coefficient test*.

*^b^Spearman’s correlation test*.

### Neutering and Cat Health

According to the feeders’ reports on the FRC population they fed, 69.7% (2325/3337) of cats were neutered and 11.8% (395/3337) were kittens; in addition, they reported that 1.6% (54/3337) of the cats were limping, 2% (67/3337) suffered from systemic disease, 4% (135/3337) had skin lesions, and 3.9% (130/3337) were cats suffering from chronic disability. Neutering rate had a significant negative association with the number of kittens and with all types of illness (Table 4).

**Table 4:** **Association of neutering rate (per feeder) with the presence of kittens and health status of 3337 reported free-roaming cats (FRC) in a survey of 222 FRC feeders in Rishon-Lezion, Israel**.

Criteria	OR	95% CI	*p*-value
Kittens	0.97	0.965–0.972	<0.001
Limping	0.99	0.977–0.995	<0.01
Systemic disease	0.98	0.969–0.985	<0.001
Skin lesions	0.99	0.986–0.998	<0.01
Chronic disability	0.99	0.985–0.996	<0.001

*Odds ratios (OR) are presented as a function of increase of 1% of neutered cats*.

## Discussion

This study provides data on the characteristics and feeding practices of FRC feeders responding to municipal TNR actions in the city of Rishon-Lezion in Israel. Although these results are from a single city, Rishon-Lezion offers a good representation of the socioeconomic status in cities located in the Dan central region, which is the most populated area in Israel (total population of 3.6 million citizens).

### Feeding Practices

We found that FRC feeders varied in the number of cats they feed, from only a few up to a few hundred cats per day. Our findings reveal that in our survey, most of the cats were fed by a small number of feeders. These “heavy” feeders, especially those who belong to feeder group 4 (who fed ≥21 FRC) are characterized by unique feeding practices and personal characteristics. In comparison to “light” feeders (feeders of groups 1–2), these feeders traveled significantly longer distances in order to feed FRC, most of them were daily feeders, delivered significantly less food per cat per day, and fed the FRC mostly with commercial dry food. These data indicate that most feeders of a large number of cats (range 21–320 cats per feeder) kept to a routine, in which they walked or even traveled by car to distinctive feeding locations on a daily basis. They delivered commercial dry food, which is more economic, constantly available, and more convenient for handling. Furthermore, the percentage of leftovers used for feeding was significantly and negatively associated with feeder group rank, in which feeders in group 1 delivered the largest percentage of leftovers. Since leftovers can be considered as the most economic but least suitable food for cats, we conclude that in comparison to “heavy” feeders (groups 3–4), “light” feeders (groups 1–2) probably invest fewer resources on average in feeding each cat.

We could not calculate daily energy delivered per cat due to the high variability of energy content in the various commercial cat diets and the unknown energy content of leftovers. However, we were able to estimate the amount of food provided to the cats according the feeders’ reports. It had been previously shown that the average food intake of high quality, *ad libitum* dry food per day per adult intact cat was 53.1–57.8 g, and for sterilized adult cats 52.0–72.8 g (16). Based on these observations and since 79% of the adult cat population in the current survey had been neutered, we could expect a range of dry food intake of approximately 52–70 g per cat per day. The mean amount of dry food delivered by “light” feeders (groups 1–2) was within the range of the expected food intake. Adding to this, the leftovers and canned moist food suggests that, as a group, these feeders deliver an amount of food that exceeds the cats’ needs. In contrast, the mean amount of dry food that was delivered by “heavy” feeders (groups 3–4) was below the expected range of food intake of these cats. As the amount of leftovers and canned moist food fed by these latter feeders was negligible, most of the FRC that were fed by “heavy” feeders had to search for additional food sources in order to complete their daily dietary requirements.

### Personal Characteristics

The tendency of older aged women who are feeding FRC was described previously in two other feeder surveys, conducted in north central Florida and Israel (12, 17). The higher prevalence of female feeders might be related to gender differences in regard to empathy toward animals, as previously suggested by Tylor and Signal (18). This suggestion is further supported by the finding of Finkler and Terkel (17) regarding the most prominent motive for caring for FRC, which is the “strong empathy toward the cats’ evident helplessness.” Owning a pet cat has been related to improved morale and decreased loneliness in women living alone (19). It is thus possible that FRC feeders and especially those of group 4 (mostly being single with less offspring and high ratio of pet cat ownership) emotionally benefit from feeding FRC similar to owners of pet cats. Despite the heavy economic burden of feeding a high number of cats, more of the “heavy feeders” (groups 3–4) were living in lower income neighborhoods, indicating the personal importance of feeding FRC for these feeders. In a survey in Israel, Finkler and Terkel (17) showed that some of the cat feeders demonstrated a high attachment level to these cats and some of them provided high levels of caretaking, regardless of their financial abilities. The data collected in this study enabled us to roughly estimate the feeding cost to the feeders. Considering the cheapest cat food (1.9$/kg) and multiplying it by the average amount of dry food that was delivered per cat per day and by the mean number of cats per feeder (see data in Table 1), we found that feeders in group 4 spent a minimum of 119$ for dry commercial food per month. This expense constituted approximately 7% of the average income in these feeders’ neighborhoods. In comparison, feeders in group 1 spent a minimum of 0.7% of the average income of their neighborhoods. These two extremes can be compared to the relative outlay on food by feeders in another study constituted by Centonze and Levy (12) in north central Florida, USA. In that survey, feeders’ financial expense constituted approximately 1% of their income.

### Neutering and Cats Health

It has previously been shown that neutered pet cats have a longer lifespan than intact cats (13, 14). Furthermore, at a UK charity neutering was found to be a protective factor for cat mortality at its adoption centers (15). However, to the best of our knowledge, to date no study has examined the effect of neutering on FRC health. In the current study neutering rate, as reported by the feeders, was negatively correlated to cat morbidity and abundance of kittens. There are several possible explanations for this association. Sexually intact adult cats are more likely to become infected with retroviruses than neutered cats (20) [though other studies found significantly higher risk for the occurrence of these diseases in adult males, compared to females, regardless of neutering status (21–23)]. Murray et al. (15) found a significant negative association between neutering rate and the cause of death of cats in adoption centers; even after excluding FIV infection, 86% of the deaths in their study were due to infectious diseases in intact cats. In addition, neutering might be indirectly associated with reduced morbidity by lowering the number of kittens. Kittens (up to 6 months old) in high density populations suffer from mortality rates ranging from 30% in neutered groups to 75% and above in intact groups (24, 25). We assume that similar to cats in catteries, high mortality rates in free-roaming kittens might be due to infectious diseases (15). This is supported by the results of a previous study performed by Nutter et al. (24) who found that most free-roaming kittens that were reported to have died during the study period showed signs of infectious diseases prior to death.

The current study population comprised feeders who had contacted the municipal veterinary services to request TNR action. We assume that other feeders may have chosen not to contact the municipality due to lack of trust of the local authorities (9), lack of knowledge of their rights, or even a lack of willingness to neuter the cats. It is possible that feeders who did contact the municipality and agreed to participate in the current survey were more prone to promoting FRC control and welfare. This potential bias might have influenced the appropriate representation of feeders from each category. Furthermore, since the current study was based on interviews, we can assume differences between cat feeders in their ability to identify, recognize, and even recall cat numbers, illness, and neutering status. However, these potential biases are not likely to affect the results of the comparison between feeder group ranks and the analysis of association between TNR and cat health, as the outcome variables should still present a random distribution between and within each group. Another limitation is the possibility for capturing individual cat more than once. We believe it is of low significance due to the large area coverage of the survey (50 km^2^, see Figure 1) and the relatively small home range known for FRC [the largest home range of intact cats found in a similar environment is 0.0075 km^2^ (26)].

Trap-neuter-return is currently the preferred method for population control and, as we have shown in this study, it also has the potential to improve FRC health. However, previous population models have predicted the limited effectiveness of those TNR programs for reducing FRC population numbers, in which the threshold of neutering percentage (approximately 75%) is not reached (27–31). Such a threshold is almost impossible to reach and sustain on a meta-population scale (such as the existing in cities in Israel). It is also evident that the availability of resources, such as food, water, and shelter, is of crucial importance in determining population size and distribution (26, 32). Furthermore, any TNR control program should involve a strict monitoring of the cat population in order to provide continuous neutering of newborn and immigrant cats (33). One possible strategy to enable both adequate monitoring and control of resources would be to locate all feedings at predefined sites. Cat feeders and especially “heavy” feeders might be incorporated in the process of planning and sustaining these predefined sites.

## Conclusion

To the best of our knowledge, this is the largest study to date to characterize feeders of FRCs and the only one to address the characteristics of extremely “heavy” cat feeders. Despite their low numbers, these feeders are dominant among the cat caretakers and their contribution to the availability of vital resources to these cats is significant. Therefore, we believe that this population of feeders should be taken into consideration in any program for managing FRC populations. Such suggested program should include predefined feeding locations, in which continuing actions of TNR will be performed. The approach of feeding in predefined locations will enable authorities to control and monitor neutering rate, cat nuisances, and cat population resources. Authorities might have the aid of devoted feeders by reporting on feeding locations (other than the predefined sites), monitoring cat populations in the predefined sites, and even assist in cat capturing during TNR campaigns. The fact that heavy feeders often travel long distances in order to feed the cats may contribute to their willingness to feed at such predefined sites. Understanding the characteristics of these “heavy” feeders may aid in understanding their incentives and motivation, and may improve communication of the authorities with them.

## Author Contributions

IG: planned the research, managed data collection, analyzed the data, and drafted the manuscript. TR: planned the research, reviewed, and participated in writing the manuscript. YZ: planned the research, involved in data collection, and reviewed the manuscript. YB: collected the data and reviewed the manuscript. EK: planned the research, analyzed the data, reviewed, and participated in writing the manuscript.

## Conflict of Interest Statement

The authors declare that the research was conducted in the absence of any commercial or financial relationships that could be construed as a potential conflict of interest.

## Abbreviations

FRC, free-roaming cats; TNR, trap-neuter-return.

## Supplement

Hi, my name is XXX and I am studying free-roaming cats in the city of Rishon-Lezion. As part of our study, we survey free-roaming cats’ feeders. This interview will last for only few minutes of your time, and its aim is to evaluate cats’ health status and numbers.

The following questions refer to your feeding habits:

1. Let us follow together to every place you use to feed the cats. For each place, please provide the following details:
  - The accurate address of feeding location (interviewer was using the city map)
  - What is the frequency you generally feed the cats in this location?
  - What is the average number of cats you feed in this ­location?
  - What type of food you generally use in this location?
  - Do you use water bowels in this location?
2. How many kilograms of dry commercial food do you buy each month?
3. How many wet commercial food do you buy each month?
4. If you feed with home-made diet, how many kilograms you feed each month?

The following questions refer to all of the cats you feed:

5. How many cats that you fed for the last 2 weeks were sterilized?
6. How many cats that you fed for the last 2 weeks were kittens up to age of 6 months?
7. How many cats that you fed for the last 2 weeks suffered from limping?
8. How many cats that you fed for the last 2 weeks suffered from a disease that diminished cat’s function, such as apathy, anorexia, and acute exercise intolerance?
9. How many cats that you fed for the last 2 weeks suffered from skin diseases, such as alopecia and wounds?
10. How many cats that you fed for the last 2 weeks suffer from permanent disability, such as eye absence, limb deformation or absence?

The following questions refer to your personal details. If you feel uncomfortable to answer, you can choose to skip some questions.

11. Man/woman (was fulfilled by the interviewer).
12. What is your current family status? Married, single, divorced, or widow?
13. Your year of birth is?
14. Your current address is?
15. Your current neighborhood?
16. Do you have offspring? Yes or no?
17. How many of your offspring live with you in house?
18. Do you own a pet inside your home? Yes or no?

If yes: how many cats? How many dogs? Any other pets?
