# Corrigendum: Feeders of Free-Roaming Cats: Personal Characteristics, Feeding Practices, and Data on Cat Health and Welfare in an Urban Setting of Israel

**DOI:** 10.3389/fvets.2020.00144

**Published:** 2020-03-31

**Authors:** Idit Gunther, Tal Raz, Yehonatan Even Zor, Yuval Bachowski, Eyal Klement

**Affiliations:** ^1^Koret School of Veterinary Medicine, Hebrew University of Jerusalem, Jerusalem, Israel; ^2^Veterinary Municipal Department, Rishon-Lezion, Israel

**Keywords:** cat feeders, cat caretakers, free-roaming cats, neutering, feeding habits, TNR, sterilization

In the original article, there was an error. The term **“siblings”** was used instead of **“offspring.”** All instances of the term **“siblings”** have now been corrected to **“offspring”** throughout the article.

The authors apologize for this error and state that this does not change the scientific conclusions of the article in any way. The original article has been updated.

